# Knowledge About Cervical Cancer and Awareness About Human Papillomavirus Vaccination Among Medical Students at the Northern Border University, Arar, Kingdom of Saudi Arabia

**DOI:** 10.7759/cureus.61313

**Published:** 2024-05-29

**Authors:** Tehreem Aftab, Ehtisham Khyzer, Nida Suhail, Hajar Abdullah Alanzy

**Affiliations:** 1 Department of Physiology, College of Medicine, Northern Border University, Arar, SAU; 2 Department of Internal Medicine, College of Medicine, Northern Border University, Arar, SAU; 3 Department of Medical Laboratory Technology, College of Applied Medical Sciences, Northern Border University, Arar, SAU; 4 Department of Medicine, Northern Border University, Arar, SAU

**Keywords:** arar, saudi arabia, medical students, cervical cancer screening, pap smear, hpv vaccine, cervical cancer, hpv infection

## Abstract

Objectives

To evaluate the knowledge and awareness about cervical cancer and human papillomavirus (HPV) vaccination among medical undergraduates at Northern Border University.

Methods

It was a cross-sectional study done on students selected conveniently from the College of Medicine, Northern Border University, Arar. The data were collected regarding knowledge about HPV infection and vaccine awareness using a validated questionnaire.

Results

A total of 200 students responded to the questionnaires, with 104 (52%) being male students and 120 (60%) being clinical years of MBBS. The mean knowledge score was 17.12 ± 2.73 out of 24, which was labeled as moderate knowledge about cervical cancer and HPV. Almost two-thirds of the students responded correctly to the etiology and risk factors of cervical cancer, while only half of the students knew the correct screening intervals for cervical cancer. The awareness of students about the HPV vaccine was deficient, and the mean score was estimated to be 4.20 ± 0.79 out of nine. Female students and students in clinical years showed significantly better understanding and awareness about cervical cancer and its vaccine and showed greater vaccine acceptability as compared to male students and students in preclinical years.

Conclusion

The present study shows moderate knowledge about cervical cancer but deficient awareness of medical students about the HPV vaccine. However, the students were willing to get educated about cervical cancer and its vaccine and showed a favorable opinion towards vaccinating the schoolgirls and educating their patients as future physicians. The information can be considered a benchmark on knowledge and awareness levels and can be utilized to modify medical curricula and develop efficient awareness programs.

## Introduction

Cervical cancer triggered by human papillomavirus (HPV) is the fourth most prevalent cancer among females. It leads to the death of almost 270,000 women annually, and most of them reside in developing countries [1]. It is responsible for 2.6% of the female cancers in Saudi Arabia and is the ninth most common cancer among females in Saudi Arabia [2]. The pathogenesis of cervical cancer may show some variation in Muslim countries, but the main risk factor is persistent HPV infection. It is the most commonly reported viral infection of the reproductive tract. It effects more than 70% of sexually active individuals at some stage in their lives. It involves the basal epithelial cells of mucocutaneous membranes and can present as benign lesions, such as warts to cancerous lesions. The infection with HPV is more prevalent among women 18-30 years of age, declining after 30 years; however, cervical cancer is commonly seen in older women, indicating the progression of chronic infection to cancer [3]. 

The prevalence of HPV infection in cervical cancer is between 85 and 99% globally [4]. HPVs are classified based on their probability of causing cervical cancer and genomic sequence into high-risk, probable high, and non-oncogenic low-risk. The infection with a high-risk HPV variant is strongly linked to the development of cervical cancer, so primary prevention can be done by using prophylactic vaccination [5]. The vaccines against HPV (bivalent and quadrivalent) stimulate a potent immune response and are safe and efficient in counteracting persistent infection and precancerous lesions in females. The HPV vaccination is considered the most economical way to decrease cancer-related morbidity and mortality and improve health outcomes [6]. According to WHO, vaccines against HPV are included in national immunization programs of almost 125 countries [7]. In Saudi Arabia, it was included in the national immunization schedule in 2017 targeting schoolgirls aged 11-12 [8]. Despite all these efforts, the acceptability and uptake of vaccines remained deficient, delaying a noticeable influence on public health. It has been shown in a study done in 2020 that only 2% of females were vaccinated in Saudi Arabia [9]. Similarly, a study done in the Eastern region has shown that only 4% of the participants received HPV vaccination [10].

It has been observed that multiple social, cultural, and religious elements lead to deficient uptake of vaccines [11]. A study done in Jizan has shown that 30% of participants refused to take the vaccine because of religious and moral reasons [12]. The hesitancy to take a vaccine due to various factors results in suboptimal coverage and negatively impacts efforts to control and reduce the burden of HPV infection and cervical cancer [13]. The most effective strategy to limit HPV infection and enhance vaccination is to improve awareness among the general population through the active participation of physicians and healthcare providers. Due to cultural and religious restraints in Saudi Arabia, it is a challenge for healthcare providers to introduce vaccines against sexually transmitted diseases to patients. Well-informed physicians can play an effective role in raising awareness about HPV vaccine safety and efficacy and facilitating women and parents to make informed decisions about HPV screening and vaccination [13]. Insufficient knowledge and awareness among physicians can be a major limiting factor in the success of vaccination and screening programs. The objective of the present study was, therefore, to assess the level of knowledge and awareness about HPV, its association with cancer, and the benefits of taking vaccines among medical students, who can play a vital role as future healthcare physicians in fostering awareness and changing community prospects.

## Materials and methods

Study design and setting

It was a cross-sectional study done on the second-year to final-year MBBS students from the College of Medicine, Northern Border University, Arar, Saudi Arabia. The study was done from March 2024 to April 2024. The Raosoft sample size calculator was used to estimate the sample size by assuming a 95% confidence level, 5% sampling error, and 50% probability of occurrence. The estimated sample size was 200. Any student who was willing to participate in study was included in study.

Data collection procedure

Data were collected using a questionnaire developed after reviewing pertinent literature. The questionnaire was pre-validated, comprising statements modified from questionnaires used in previous studies [14,15]. A pilot study was done on 30 participants to assess the clarity and remove any ambiguous statements. 

The questionnaire comprised three parts. The first part collected information concerning demographic details like age, gender, academic year, and academic scores. The second portion was designed to assess the knowledge of students regarding HPV infection and its relationship with cervical cancer. The third part consisted of questions related to awareness and acceptability of the HPV vaccine.

The knowledge of students regarding cervical cancer and HPV was assessed by 11 questions with 24 statements related to etiology, risk factors, methods of transmission, means of prevention, clinical features, screening methods, and screening interval. The students responded by selecting correct, incorrect, or don’t know options. Each correct answer related to knowledge was given a credit of 1 or 0 for incorrect or don’t know answers. The knowledge score for HPV infection ranged from 0 to 24. The students were categorized as having poor, moderate, and good knowledge if the scores were 0-12, 13-17, and 18-24, respectively. At the end of this section, they were asked about the sources of their knowledge about cervical cancer and HPV infection. The third section of the questionnaire evaluated students' awareness and acceptability of the HPV vaccine. It contained nine true/false and don’t know questions related to vaccine availability, dose of vaccine, the specific age group for vaccine administration and its protective role against cervical cancer. Each right response was given a credit of one. A score of seven out of nine indicated a good awareness score, while a score below five was considered a poor score. In the last segment of the questionnaire, the student's vaccine acceptability was assessed. They were asked whether they were interested in taking the vaccine and, if not, what the reasons were that triggered them to do so. They were asked about the role of education programs in motivating people to get the vaccine and whether they would recommend the vaccine as a future healthcare provider. 

Ethical considerations

The study was done after obtaining ethical approval from the Local Bioethics Committee at Northern Border University. Informed consent was taken from students before filling out the questionnaires. The study was conducted in accordance with the guidelines of the Helsinki Declaration.

Data analysis

The data analysis was done using IBM SPSS Statistics for Windows, Version 20 (released 2011; IBM Corp., Armonk, New York, USA). Categorical variables were represented as frequency and percentage, while mean and standard deviation were estimated for continuous data. Categorical variables were evaluated by the chi-square test, while the student t-test was utilized to compare the mean between the two groups. The Cronbach’s alpha coefficient with a value ≥0.70 was considered acceptable for the questionnaire. A p-value <0.05 was labeled as statistically significant. 

## Results

The study included 200 medical undergraduates with a mean age of 25.12 ± 1.42. There were 104 (n = 52%) male students and 96 (42%) female students. Most of the students were in clinical years of MBBS 120 (60%). The information regarding cervical cancer and its vaccine was obtained mainly from the internet 64 (32%), followed by education 48 (24%), and colleagues/friends 40 (20%), as shown in Table 1.

**Table 1:** Characteristics of study participants.

	Frequency (n)	Percent (%)
Age (mean ± SD)	25.12 ± 1.42	
Gender		
Male	104	52
Female	96	48
Year of study		
Preclinical	80	40
Clinical	120	60
Source of information		
Education/curriculum	48	24
Internet	64	32
Colleagues and friends	40	20
Self-learning	20	10
Health professionals	20	10
Miscellaneous	8	4

Knowledge of students regarding cervical cancer

Knowledge of students regarding cervical cancer was assessed by asking 11 questions with a total of 24 points regarding etiology, risk factors, clinical features, and preventive and screening methods. The mean knowledge score about cervical cancer and HPV was 17.12 ± 2.73. Table 2 shows the correct responses of students related to cervical cancer and HPV. Most of the students were aware that HPV is the leading cause for cervical cancer 149 (74.5%) and its subtypes 6 and 11 lead to genital warts 142 (71%) and infection with HPV 16 and 18 predisposes to cervical cancer 130 (65%). The students knew that having multiple sexual partners 160 (80%), long-term OCP use 144 (72%), poor hygiene 158 (79%), and intercourse at a young age 156 (78%) makes the person more susceptible to cervical cancer. The majority of the students knew that cervical cancer can be prevented 189 (94.5%) and that vaccines play an essential role in prevention 184 (92%). However, only half of the students were aware about the correct mode of transmission and screening intervals for cervical cancer 106 (53%) and falsely considered fever 112 (56%) and itching 105 (52.5%) as the main clinical features for cervical cancer.

**Table 2:** Knowledge of students about cervical cancer and human papillomavirus. OCPs: oral contraceptive pills, PCR: polymerase chain reaction, HPV: human papillomavirus.

	Questions	Correct responses, n (%)	Wrong and don't know responses, n (%)
1.	Etiology	149 (74.5)	51 (25.5)
2.	Modes of transmission	102 (51)	98 (49)
3.	Diagnosed in the early stages	120 (60)	60 (30)
4	HPV 6 and 11 are associated with genital warts	142 (71)	58 (29)
5	HPV 16 and 18 are associated with cervical cancer	130 (65)	70 (35)
6	Risk factors		
	Multiple sexual partners	160 (80)	40 (20)
	Long term OCPs	144 (72)	56 (28)
	Poor hygiene	158 (79)	42 (21)
	Early intercourse	156 (78%)	44 (22)
7	Prevention of cervical cancer	189 (94.5)	11 (5.5)
8	Preventive methods		
	Vaccine	184 (92)	16 (8)
	Barrier method	176 (88)	24 (12)
	Single partner	189 (94.5)	11 (5.5)
	Personal hygiene	176 (88)	24 (12)
9	Recommended screening intervals (screening every three years for 25-44 years age group and every five years for women aged 45–60 years).	106 (53)	94 (47)
10	Clinical features		
	Discharge per vagina	144 (72)	56 (28)
	Lower back pain	98 (49)	102 (51)
	Anemia	85 (42.5)	115 (57.5)
	Fever (no)	88 (44)	112 (56)
	Itching (no)	95 (47.5)	105 (52.5)
11	Techniques available for HPV detection		
	Pap smear	135 (67.5)	65 (32.5)
	PCR	148 (74)	52 (26)
	Biopsy	166 (83)	34 (17)
	Blood	70 (35)	130 (65)
	Mean knowledge score about cervical cancer and HPV	17.12 ± 2.73	

Awareness of students about the cervical cancer vaccine

The mean awareness score for the HPV vaccine was 4.20 ± 0.79, with a range of 0-9 (Table 3). Almost half of the students, 104 (52%), knew that the HPV vaccine is available in Saudi Arabia, but only 56 (28%) were aware about the correct age group (11-29 years) and number of doses required for the HPV vaccine. There were 48 (24%) students who knew that it could be given to boys as well. Almost 128 (64%) correctly responded that the vaccine can be given to persons already infected with HPV and screening is not required prior to vaccination 72(36%). The vaccine that provided 70% protection against cervical cancer was answered correctly by 104 (52%) of the respondents. There were 120 (60%) students who accepted the idea that it is necessary to encourage and vaccinate schoolgirls in Saudi Arabia (Table 3).

**Table 3:** Awareness of students regarding the cervical cancer vaccine. HPV: human papillomavirus, KSA: Kingdom of Saudi Arabia.

	Questions	Correct responses, n (%)	Wrong and don’t know responses, n (%)
1.	Are vaccines available in KSA?	104 (52)	96 (48)
2.	Which age group vaccines should be given?	56 (28)	144 (72)
3.	Can vaccine be given to boys?	48 (24)	152 (76)
4	Number of doses required	56 (28)	144 (72)
5.	Can it be given to infected persons?	128 (64)	72 (36)
6.	Can it be given to sexually active person?	112 (56)	88 (44)
7.	Is screening required before vaccination?	72 (36)	128 (64)
8.	Cervical cancer protection provided by vaccine	104 (52)	96 (48)
9.	Vaccine acceptability	120 (60)	80 (40)
Mean awareness score about HPV vaccine		4.20 ± 0.79	

Knowledge and awareness scores about HPV

The knowledge and awareness scores were significantly high among female students, students in clinical years, and those residing in urban areas, as indicated in Table 4. There were 130 (65%) students who were willing to get vaccinated and supported the idea of vaccinating schoolgirls in Saudi Arabia. The relation of participant characteristics with vaccine acceptance was evaluated using the chi-square test. Female students (p-value = 0.004) and students in clinical years (p-value = 0.002) showed significantly higher levels of acceptance as compared to male students and students in preclinical years.

**Table 4:** Knowledge and awareness scores about HPV and vaccine acceptance classified by participants' characteristics. HPV: human papillomavirus.

Parameter			Knowledge score		Awareness score		Vaccine acceptance		
		N (%)	Mean (SD)	P-value	Mean (SD)	P-value	Yes %	No %	P-value
Gender	Male	104 (52)	16.23 (2.95)	0.003	4.02 (0.67)	0.001	58 (29)	46 (23)	0.004
	Female	96 (48)	18.08 (2.11)		4.39 (0.87)		72 (36)	24 (12)	
Year of study	Preclinical	80 (40)	16.70 (3.21)	0.038	4.00 (0.79)	0.001	62 (31)	18 (9)	0.002
	Clinical	120 (60)	17.40 (2.34)		4.34 (0.79)		68 (34)	52 (26)	
Place of study	Rural	96 (48)	16.41 (3.24)	<0.01	4.01 (0.76)	<0.001	68 (34)	28 (14)	0.097
	Urban	104 (52)	17.76 (1.97)		4.38 (0.79)		62 (31)	42 (21)	
Know someone with cancer	Yes	68	16.42 (3.04)	0.066	4.29 (0.83)	0.109	54 (27)	25 (12.5)	0.422
	No	132	17.47 (2.50)		4.14 (0.77)		76 (38)	45 (22.5)	

**Figure 1:**
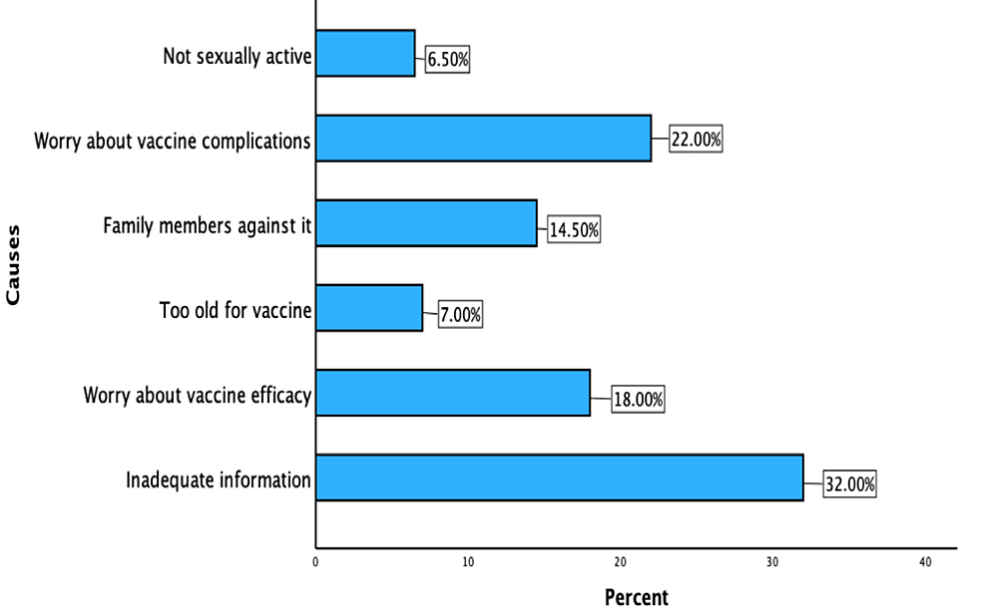
Causes of vaccine refusal.

The main cause for vaccine refusal among students was inadequate information about the vaccine (32%), followed by worry about vaccine complications (22%) and its efficacy (18%), as shown in Figure 1.

## Discussion

The present study indicates that medical students of Northern Border University have moderate knowledge about cervical cancer and HPV. The majority of the participants in the study knew that cervical cancer is precipitated by a viral infection from specific subtypes of HPV. The results are similar to the findings of other studies conducted on healthcare providers [9,16]. The students correctly recognized multiple sexual partners, young age at first intercourse, and contraceptives as the precipitating factors for cervical cancer consistent with the findings of other studies. However, knowledge regarding clinical features, diagnostic tests, and screening intervals was deficient. The majority of students recognized fever and itching of the cervix as the presenting complaints instead of anemia and lower back pain. Only one-third of students correctly recognized the PCR as a diagnostic test and recommended screening intervals for females. Similar findings are reported by a study done on medical students of Jordan showing insufficient knowledge about risk factors and screening [17]. The knowledge scores were significantly higher among females and medical students in clinical years, as compared to males and preclinical years, consistent with the results of previous literature [18,19]. 

The worldwide availability of the HPV vaccine is a great invention to limit the spread of cervical cancer. Many studies have been done previously to assess knowledge and awareness about vaccines. The current study indicates that only half of the medical students, 104 (52%), knew about the availability of vaccines in Saudi Arabia. A study done on medical students in Jordan has shown 40% awareness about the availability of vaccines in Jordan [17]. The overall awareness score about the HPV vaccine was 4.20 ± 0.79 indicating deficient awareness. Similar low scores have been reported by a study done in India [20]. There were only 48 (24%) students who knew that vaccines could be given to boys and only 56 (28%) knew that the correct number of doses required is three indicating insufficient knowledge about vaccines. A cross-sectional study conducted globally among medical students has shown that American and European students had better knowledge about cervical cancer and its vaccine as compared to African and Asian students [21]. These results are consistent with the findings of our study as well as studies conducted in India and China [22,23].

The present study reported that 120 (60%) of students supported the idea of vaccinating schoolgirls and were willing to recommend vaccination to their patients as future physicians. Similar results have been reported by Hoque et al., showing vaccine acceptance among physicians [24]. The main reasons for vaccine refusal identified by the students were a lack of complete knowledge about HPV and its vaccine, followed by fear of complications and vaccine efficacy. Similar reports about vaccine refusal have been identified in other studies. Therefore, educational campaigns and workshops should be organized to educate future healthcare providers and improve their communication skills to motivate the public to get vaccinated [25].

The current study identifies the knowledge gaps that can help in designing future educational programs and developing structured curricula. Studies have shown a significant association between modified educational strategies and improved understanding and awareness of HPV among healthcare providers [26]. 

The study has a few limitations. The main limitation of the study was the small sample size, and only medical students were sampled, so the results cannot be generalized to all university students in Saudi Arabia. Secondly, participation in the study was voluntary, so students who responded to questionnaires may be more knowledgeable and aware about health in general. This could have resulted in selection bias and overestimation of outcomes. Further multicenter studies and studies targeting to assess awareness among young students are recommended.

## Conclusions

The present study indicates insufficient knowledge and awareness about cervical cancer and its vaccine among medical undergraduates. Even though the awareness scores were low, students were willing to get educated about HPV and its vaccine. It is imperative to modify the medical curriculum, organize workshops and seminars to enhance the knowledge of students who are future healthcare providers. These future physicians, in turn, can educate their patients, address religious and cultural concerns to enhance vaccine acceptability and its use.

## References

[1] Sung H, Ferlay J, Siegel RL, Laversanne M, Soerjomataram I, Jemal A, Bray F (2021). Global cancer statistics 2020: GLOBOCAN estimates of incidence and mortality worldwide for 36 cancers in 185 countries. CA Cancer J Clin.

[2] Faqih L, Alzamil L, Aldawood E (2023). Prevalence of human papillomavirus infection and cervical abnormalities among women attending a tertiary care center in Saudi Arabia over 2 years. Trop Med Infect Dis.

[3] Hussain AN, Alkhenizan A, McWalter P, Qazi N, Alshmassi A, Farooqi S, Abdulkarim A (2016). Attitudes and perceptions towards HPV vaccination among young women in Saudi Arabia. J Fam Commun Med.

[4] Scott-Wittenborn N, Fakhry C (2021). Epidemiology of HPV related malignancies. Semin Radiat Oncol.

[5] Athanasiou A, Bowden S, Paraskevaidi M, Fotopoulou C, Martin-Hirsch P, Paraskevaidis E, Kyrgiou M (2020). HPV vaccination and cancer prevention. Best Pract Res Clin Obstet Gynaecol.

[6] Loke AY, Kwan ML, Wong YT, Wong AK (2017). The uptake of human papillomavirus vaccination and its associated factors among adolescents: a systematic review. J Prim Care Commun Health.

[7] Ebrahimi N, Yousefi Z, Khosravi G (2023). Human papillomavirus vaccination in low- and middle-income countries: progression, barriers, and future prospective. Front Immunol.

[8] (2024). Immunization schedule: Ministry of Health, 2018. https://www.moh.gov.sa/en/HealthAwareness/EducationalContent/vaccination/Pages/vaccination1.aspx.

[9] Almazrou S, Saddik B, Jradi H (2020). Knowledge, attitudes, and practices of Saudi physicians regarding cervical cancer and the human papilloma virus vaccine. J Infect Public Health.

[10] Almaghlouth AK, Bohamad AH, Alabbad RY, Alghanim JH, Alqattan DJ, Alkhalaf RA (2022). Acceptance, awareness, and knowledge of human papillomavirus vaccine in Eastern Province, Saudi Arabia. Cureus.

[11] Gallagher KE, LaMontagne DS, Watson-Jones D (2018). Status of HPV vaccine introduction and barriers to country uptake. Vaccine.

[12] Darraj AI, Arishy AM, Alshamakhi AH (2022). Human papillomavirus knowledge and vaccine acceptability in Jazan province, Saudi Arabia. Vaccines (Basel).

[13] Aldawood E, Dabbagh D, Alharbi S, Alzamil L, Faqih L, Alshurafa HH, Dabbagh R (2023). HPV vaccine knowledge and hesitancy among health colleges' students at a Saudi University. J Multidiscip Healthc.

[14] Singh J, Baliga SS (2021). Knowledge regarding cervical cancer and HPV vaccine among medical students: a cross-sectional study. Clin Epidemiol Glob Health.

[15] Farsi NJ, Baharoon AH, Jiffri AE, Marzouki HZ, Merdad MA, Merdad LA (2021). Human papillomavirus knowledge and vaccine acceptability among male medical students in Saudi Arabia. Hum Vaccin Immunother.

[16] Sherman SM, Bartholomew K, Denison HJ, Patel H, Moss EL, Douwes J, Bromhead C (2018). Knowledge, attitudes and awareness of the human papillomavirus among health professionals in New Zealand. PLoS One.

[17] Alsous MM, Ali A, Al-Azzam S, Karasneh R, Amawi H (2021). Knowledge about cervical cancer and awareness about human papillomavirus vaccination among medical students in Jordan. PeerJ.

[18] Ngwenya D, Huang SL (2018). Knowledge, attitude and practice on cervical cancer and screening: a survey of men and women in Swaziland. J Public Health (Oxf).

[19] Tesfaye ZT, Bhagavathula AS, Gebreyohannes EA, Tegegn HG (2019). Knowledge and awareness of cervical cancer and human papillomavirus among female students in an Ethiopian University: a cross-sectional study. Int J Prev Med.

[20] Gollu AN, Gore CA (2021). Knowledge, awareness and attitude of medical students regarding HPV infection and HPV vaccination. Asian Pac J Cancer Care.

[21] Gismondi M, Augustine AM, Tahir Khokhar MA, Khokhar HT, Twentyman KE, Florea ID, Grigore M (2021). Are medical students from across the world aware of cervical cancer, HPV infection and vaccination? A cross-sectional comparative study. J Cancer Educ.

[22] Patel IS, Dongara AR, Mungala BM, Chapla A, Phatak AG, Nimbalkar SM (2021). Knowledge and attitude about cervical cancer and human papillomavirus vaccine among medical and paramedical students of a university. J Fam Med Prim Care.

[23] Wen Y, Pan XF, Zhao ZM (2014). Knowledge of human papillomavirus (HPV) infection, cervical cancer, and HPV vaccine and its correlates among medical students in Southwest China: a multi-center cross-sectional survey. Asian Pac J Cancer Prev.

[24] Hoque ME (2016). Factors influencing the recommendation of the human papillomavirus vaccine by South African doctors working in a tertiary hospital. Afr Health Sci.

[25] Hswen Y, Gilkey MB, Rimer BK, Brewer NT (2017). Improving physician recommendations for human papillomavirus vaccination: the role of professional organizations. Sex Transm Dis.

[26] Makadzange EE, Peeters A, Joore MA, Kimman ML (2022). The effectiveness of health education interventions on cervical cancer prevention in Africa: a systematic review. Prev Med.

